# Polycystin-1 regulates tendon-derived mesenchymal stem cells fate and matrix organization in heterotopic ossification

**DOI:** 10.1038/s41413-024-00392-y

**Published:** 2025-01-20

**Authors:** Yi Li Xu, Mei Huang, Yang Zhang, Xin Ying Su, Min Huang, Nan Yu Zou, Yu Rui Jiao, Yu Chen Sun, Ling Liu, Yong Hua Lei, Chang Jun Li

**Affiliations:** 1 Department of Endocrinology, Endocrinology Research Center, Xiangya Hospital of Central South University, Changsha, Hunan 410008 China; 2 Department of Orthodontics, Xiangya Hospital, Central South University, Changsha, Hunan Province China; 3 Xiangya School of Medicine, Central South University, Changsha, Hunan Province 410013 China; 4 Key Laboratory of Aging-related Bone and Joint Diseases Prevention and Treatment, Ministry of Education, Xiangya Hospital, Central South University, Changsha, China; 5 National Clinical Research Center for Geriatric Disorders, Xiangya Hospital, Central South University, Changsha, Hunan 410008 China; 6 Laboratory Animal Center, Xiangya Hospital, Central South University, Changsha, Hunan 410008 China

**Keywords:** Pathogenesis, Bone

## Abstract

Mechanical stress modulates bone formation and organization of the extracellular matrix (ECM), the interaction of which affects heterotopic ossification (HO). However, the mechanically sensitive cell populations in HO and the underlying mechanism remain elusive. Here, we show that the mechanical protein Polysyctin-1 (PC1, *Pkd1*) regulates CTSK lineage tendon-derived mesenchymal stem cell (TDMSC) fate and ECM organization, thus affecting HO progression. First, we revealed that CTSK lineage TDMSCs are the major source of osteoblasts and fibroblasts in HO and are responsive to mechanical cues via single-cell RNA sequencing analysis and experiments with a lineage tracing mouse model. Moreover, we showed that PC1 mediates the mechanosignal transduction of CTSK lineage TDMSCs to regulate osteogenic and fibrogenic differentiation and alters the ECM architecture by facilitating TAZ nuclear translocation. Conditional gene depletion of *Pkd1* or *Taz* in CTSK lineage cells and pharmaceutical intervention in the PC1-TAZ axis disrupt osteogenesis, fibrogenesis and ECM organization, and consequently attenuate HO progression. These findings suggest that mechanically sensitive CTSK-lineage TDMSCs contribute to heterotopic ossification through PC1-TAZ signaling axis mediated cell fate determination and ECM organization.

## Introduction

Heterotopic ossification (HO) is a debilitating pathological process that impairs the mobility of patients through the formation of abnormal bone in soft tissues, especially near joints, following trauma or burn injuries.^1–3^ In response to inflammatory triggers, certain cytokines or extracellular matrix alterations in the microenvironment induce stem cells that are programmed to become soft tissue to alter their fate and acquire an osteogenic commitment, resulting in heterotopic ossification.^3–5^ This series of pathological changes causes difficult clinical symptoms, such as ectopic ossification, which can cause pain, and joint movement limitations in severe cases.^6,7^ Several studies have reported that immobilization is an effective conservative treatment for reducing HO based on clinical data, but the underlying mechanism is not fully understood. This may be due to alterations in the fibroblast-woven extracellular matrix (ECM), which influences osteogenic activity.^8^ Research has revealed that immobilization intervention changes the ECM structure of the injured area and causes mesenchymal stem cells to undergo fate bias.^4^ Moreover, mesenchymal lineage cells in the injured area also interact with the surrounding ECM and change the ECM structure through collagen-binding proteins.^9^ Importantly, the mechanical stress required for bone formation and ECM organization affected by immobilization intervention. However, the role of mechanical stress in HO, in which these cells sense mechanical cues and how these cells sense changes in mechanical stress and subsequently alter cell fate and ECM organization to affect HO initiation and progression needs to be further defined.

Previous studies have shown that various types of cells, such as Achilles tendon stem cells, mesenchymal stem cells, and even pericytes, may contribute to HO formation.^10–12^ However, the specific type of stem cell that participates in the interactions between aberrant osteogenic activity and the ECM remains unclear. This study revealed that mechanosensitive CTSK-lineage tendon-derived mesenchymal stem cells with unique osteogenic and fibrogenic potential influence HO formation and ECM remodeling in an Achilles tendon injury mouse model. CTSK has been shown to label not only osteoclasts and periosteum stem cells but also a fraction of tendon-derived progenitor cells.^13^ Enhancing the osteogenic activity of CTSK^+^ cells via gene mutation of Hedgehog signaling resulted in spontaneous heterotopic ossification.^13^ However, the properties and multipotency of CTSK^+^ tendon-derived mesenchymal stem cells at musculoskeletal injury sites remain elusive. In this study, we observed an increase in the number of CTSK-lineage tendon-derived mesenchymal stem cells, which underwent osteogenic and fibrogenic differentiation with mechanical stimulation after injury. Immobilization alleviated local tissue stress, attenuated the osteogenic and fibrogenic differentiation of CTSK-lineage tendon-derived mesenchymal stem cells, and disrupted ECM organization.

Mechanical forces exert biological effects through various mechanotransduction mechanisms. Polycystin-1 (PC1) is a known transmembrane mechanosensory protein that forms complexes to sense fluid shear stress in renal epithelial cells via its coiled-coil domain interaction.^14^ PC1 activates the osteogenic gene *Runx2* by releasing its C-terminal tail (CTT) and thereby stimulating the transcriptional coactivator TAZ.^15^ A previous study demonstrated that BMSCs sense mechanical stimulation through the PC1-TAZ pathway to promote osteogenesis and inhibit adipogenesis.^16^ Additionally, the PC1-TAZ axis mediates conductive mechanical stimulation in osteoblasts.^17^ A previous study showed that TRPV4, a calcium channel mechanoreceptor protein, regulates ECM alignment.^18^ However, the role of PC1 in ectopic ossification and ECM organization is unclear.

Here, we discovered that CTSK lineage tendon-derived mesenchymal stem cells (TDMSCs) detect mechanical cues in the injured Achilles tendon through the PC1-TAZ mechanotransduction axis. After immobilization, the osteogenic and fibrogenic potential of CTSK-lineage TDMSCs was impaired. ScRNA analysis indicated that the PC1-TAZ axis was a key mechanistic signal in this process. Deletion of the *Pkd1* or *Taz* genes in CTSK^+^ cells in vivo impaired their osteogenic and fibrogenic differentiation capacity and affected the ECM arrangement. DAPT, a PC1-TAZ pathway inhibitor, effectively reduced osteogenic and fibrogenic activity after injury. In conclusion, these findings suggest that mechanical stress regulates MSC fate and ECM formation via the PC1-TAZ axis, thus affecting HO formation and progression.

## Results

### CTSK-lineage tendon-derived mesenchymal stem cells are skewed toward osteoblastic and fibroblastic lineage cells after Achilles tendon injury

**Fig. 1:**
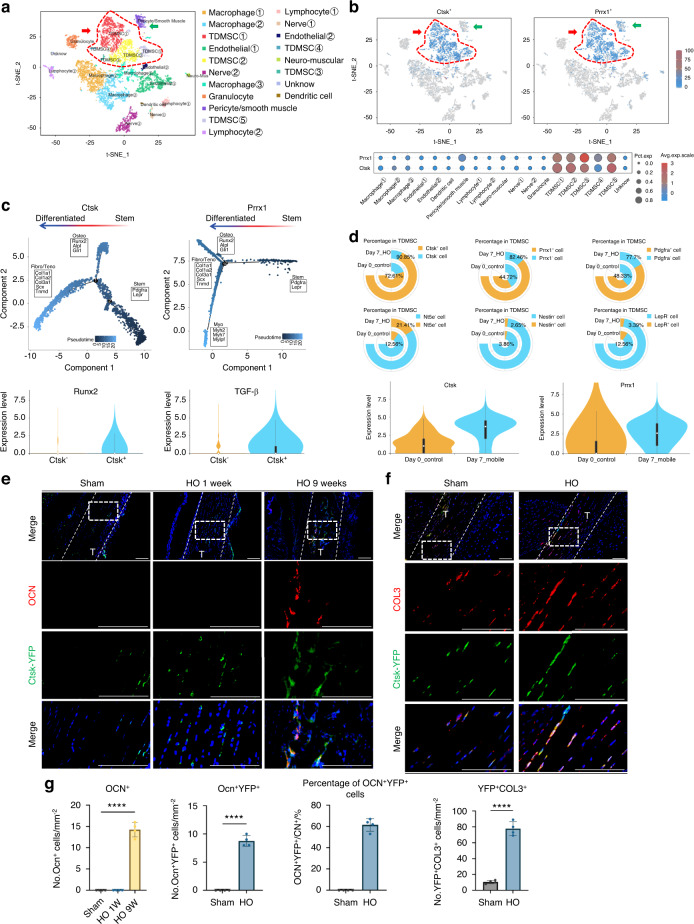
CTSK lineage tendon-derived mesenchymal stem cells differentiate into osteogenic and fibrogenic directions at the Achilles tendon site after injury. **a** Single-cell sequencing data analysis of the Achilles tendon injury site, identifying 19 clusters, including mesenchymal stem cells based on *Pdgfra* and *Prrx1* genes, where the cell population with higher expression of these two genes is defined as TDMSCs, macrophage based on *Lyz2* and *Cd68*, etc. **b** Expression distribution of *Ctsk*^*+*^ and *Prrx1*^*+*^ cells in all the cells obtained by single-cell sequencing. **c** Trajectory analysis of gene expression changes across pseudotime in the *Ctsk*^*+*^ and *Prrx1*^+^cell cluster. **d** Cell frequency analysis of several representative marker genes in the TDMSC cell population, including *Ctsk*, *Prrx1*, *Pdgfra*, *Nt5e*, *Nestin* and *LepR*. **e** Representative images of immunofluorescence staining with anti-GFP and anti-OCN. Scale bars: 100μm. Cell nuclei were stained with DAPI. The Achilles tendon images at the HO site (top) were marked by white dashed lines at x20 magnification, and the images at x63 magnification (bottom) in the white dashed boxes were selected for statistical analysis of cell counting, showing the antibody staining channels separately. **f** Representative images of immunofluorescence staining with anti-GFP and anti-COL3 in the specimens of CTSK-traced mice 1 week after injury modeling. Scale bars: 100 μm. **g** Statistical comparison of the number of OCN^+^ cells, YFP^+^OCN^+^ and YFP^+^ COL3^+^ double-positive cells in the field of view in the images at x63 magnification. (*n* = 4/group, ****P < 0.000 1)

To identify the phenotype of aberrantly differentiated MSCs after injury, we performed unbiased single-cell sequencing data analysis of the entire local tissue after Achilles tendon injury modeling (GEO: GSE150995). We classified mesenchymal cells with higher expression of *Prrx1* and *Pdgfrα* which have stronger stem cell characteristics, as TDMSCs (Fig. S1A). The results of the cell fractionation t-SNE plots for single-cell analysis are displayed in Fig. 1a. Compared with the uninjured area, the number of TDMSCs in the injured area significantly increased after injury-induced heterotopic ossification (HO) (Fig. S2A,B). We found that *Ctsk* was specifically expressed in mesenchymal stem cells in the Achilles tendon area than was the classical mesenchymal stem cell marker *Prrx1*, the expression of which was also detected in other types of cells, including perivascular cells and smooth muscle cells (Fig. 1b). The CTSK^+^ cells covered a large portion of the tendon-derived mesenchymal stem cells (Fig. 1b). This remains the case in the late stages of injury (Fig. S1B). At the same time, pseudotime analysis of the CTSK^+^ cell population found that CTSK^+^ cells could differentiate into osteogenic and fibrogenic directions after injury (Fig. 1c). Pseudotime analysis of the PRRX1^+^ cell population found that, in addition to osteogenic and fibroblastic differentiation, PRRX1^+^ cells also differentiated toward myogenesis (Fig. 1c). Cell frequency analysis of several representative marker genes in the MSC population revealed that CTSK^+^ cells were more injury-responsive and widely distributed (Fig. 1d, Fig. S1E). Not only was the number of CTSK^+^ cells increased, but their overall expression was also greater than prrx1. In addition, we compared signature target genes in the osteogenic and fibrotic directions and found that the expression of osteogenic and fibrotic signature genes was significantly greater in CTSK^+^ cells than in CTSK^-^ cells (Fig. 1d, Fig. S1D). We also found that the CTSK^+^ gene labeled more cells than TDPCs which just expressing tendon-specific genes (Fig. S1C, F).

To further study the function and fate of CTSK lineage mesenchymal cells, we constructed transgenic CTSK-YFP lineage tracing mice with the HO mouse model (Fig. S1G, H). Micro-CT scanning and Masson’s trichrome staining revealed ectopic bone formation and bone marrow cavity in the Achilles tendon area (Fig. S1I, J). Further immunofluorescence staining revealed that many CTSK-YFP cells were costianed with osteocalcin (OCN, an osteoblast marker), indicating that CTSK^+^ cells or their subsequent differentiated cells formed osteogenic cells (Fig. 1e, g). To further study the differentiation of CTSK^+^ lineage cells into fibroblasts in situ, COL3^+^ cells, which represent newly formed fibers, were measured and showed costained with CTSK^+^ lineage cells in the injured Achilles tendon area (Fig. 1f, g), which revealed that CTSK^+^ cells can differentiate into COL3^+^ fibroblasts upon injury. These data from a lineage tracing mouse model and single-cell sequencing data suggest that CTSK- lineage TDMSCs are amajor source of oste oblastic and fibroblastic lineage cells after Achilles tendon injury.

### The CTSK lineage tendon-derived mesenchymal stem cell population is sensitive to mechanical stimulation

**Fig. 2:**
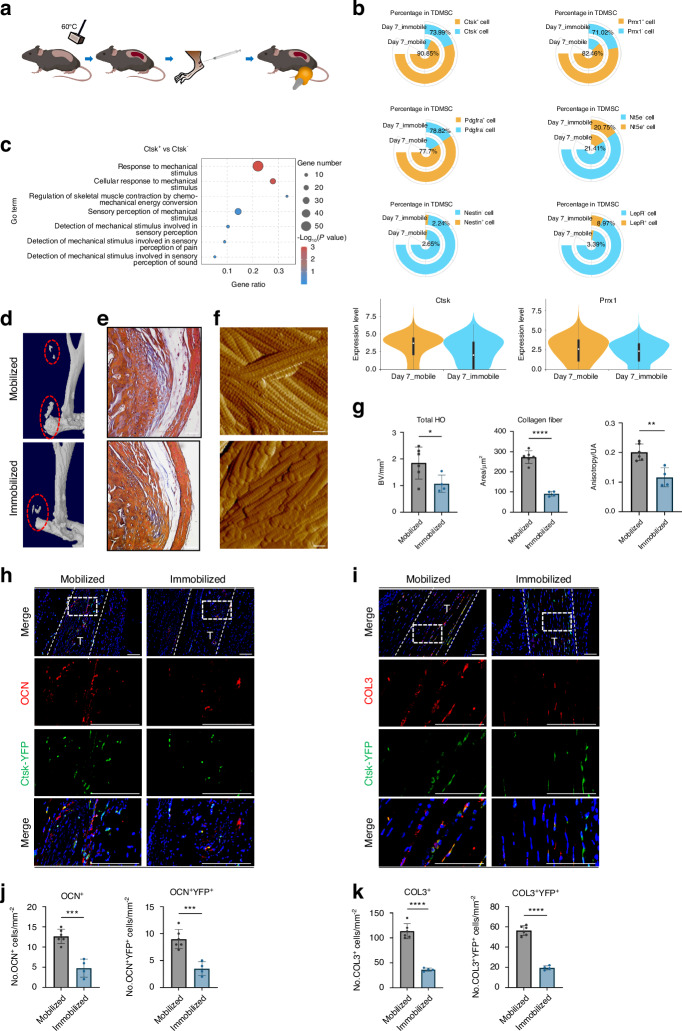
CTSK lineage tendon-derived mesenchymal stem cell population is sensitive to mechanical stimulation. **a** Schematic diagram of immobilized mechanical intervention after Achilles tendon injury modeling by Burn/ATP. **b** Cell frequency analysis of several representative marker genes in the TDMSC cell population comparing Mobile and Immobile, including Ctsk, Prrx1, Pdgfra, Nt5e, Nestin and LepR. **c** GO analysis of *Ctsk*^*+*^ as well as *Ctsk*^*-*^ cell populations revealed alterations in several mechanical force-related pathways. **d**, **e** Mice were subjected to mobile and immobile interventions after Burn/ATP modeling, and specimens were collected for MicroCT scanning, followed by decalcification and paraffin embedding of the specimens for masson immunohistochemical staining. Scale bars: 100 μm. **f** Atomic force microscopy scanning images of the ECM arrangement of the specimens of the mobile and immobile groups after injury. Scale bars: 1 μm. **g** Statistical analysis results of the bone volume of ectopic ossification shown by MicroCT scanning. Statistical analysis of the area of collagen fibers in the field of view of the masson immunohistochemical staining microscope images and Anisotropy analysis of AFM images of the mobile and immobile groups by independent samples *t*-test (*n* = 6/group, *P < 0.05, **P < 0.01, ****P < 0.000 1). **h** Representative images of immunofluorescence staining with anti-GFP and anti-OCN of the specimens of Ctsk-YFP lineage-traced mice divided into mobile and immobile groups after Burn/ATP modeling. Scale bars: 100 μm. **i** Representative images of immunofluorescence staining with anti-GFP and anti-COL3 of the specimens of Ctsk-YFP lineage-traced mice divided into mobile and immobile groups after Burn/ATP modeling. Scale bars: 100 μm. **j** Statistical comparison of the number of OCN^+^ and YFP^+^ cells in the field of view of the images, comparing the mobile and immobile groups by independent samples *t*-test (mobile group *n* = 6, immobile group *n* = 4, ***P < 0.001). **k** Statistical comparison of the number of COL3^+^ cells and COL3^+^YFP^+^ double-positive cells in the field of view of the images, comparing the mobile and immobile groups by independent samples *t*-test (mobile group n = 6, immobile group *n* = 4, ***P < 0.001)

To study the effects of mechanical stimulation on HO, we immobilized the distal limbs of the mice after Achilles tendon injury, so that the ankle joints of the mice were in plantar flexion (Fig. 2a). This fixed position placed the Achilles tendon and triceps surae in a relaxed state and under nonstretching stress. Moreover, the external immobilization device prevented pressure from being transmitted to the ankle joint when the mouse walked.^19^ We performed single-cell sequencing data analysis of the Achilles tendon area of mobile and immobile mice after injury modeling and found that with immobilizaztion, the CTSK lineage cell population was decreased compared with that in the mobile group (Fig. 2b, Fig. S2C). Additionally, CTSK^+^ TDMSCs were a more mechanosensitive and widely distributed cell subpopulation. A comparison of mobile and immobile samples also revealed that CTSK expression levels changed considerably with mechanical conditions (Fig. 2b, Fig. S2D). The GO analysis of the CTSK^+^ and CTSK^-^ cell populations revealed alterations in several mechanical force-related pathways (Fig. 2c).

To further verify the effect of mechanical stimulation on CTSK lineage cell function in vivo, we performed immobilized interventions on a CTSK-YFP lineage tracing mouse model after Burn/Achilles tendon puncture modeling. The volume of heterotopic ossification decreased after immobilization (Fig. 2d, g), and the number of collagen fibers at the connection between the Achilles tendon and calcaneus also decreased (Fig. 2e, g). Moreover, although the ultrastructure of the ECM appeared to be unaltered, the arrangement of the ECM became more disorganized after immobilization (Fig. 2f, g). We performed an in vitro tensile stress test on isolated TDMSCs and obtained similar results, with enhanced osteogenic and fibroblastic differentiation following stress. Immunofluorescence staining analysis revealed that the number of CTSK^+^ cell-derived osteoblast-like cells decreased after immobilization (Fig. 2h, j). To detect fibrogenic activity in the Achilles tendon area under different mechanical intervention conditions after injury, we performed immunofluorescence staining of newly formed fibrous COL3 and found that the number of cells expressing CSTK and COL3 decreased after immobilization (Fig. 2i, k).

### Polycystin-1 mediated mechanical signals affect abnormal osteogenesis and ECM structures after Achilles tendon injury

**Fig. 3:**
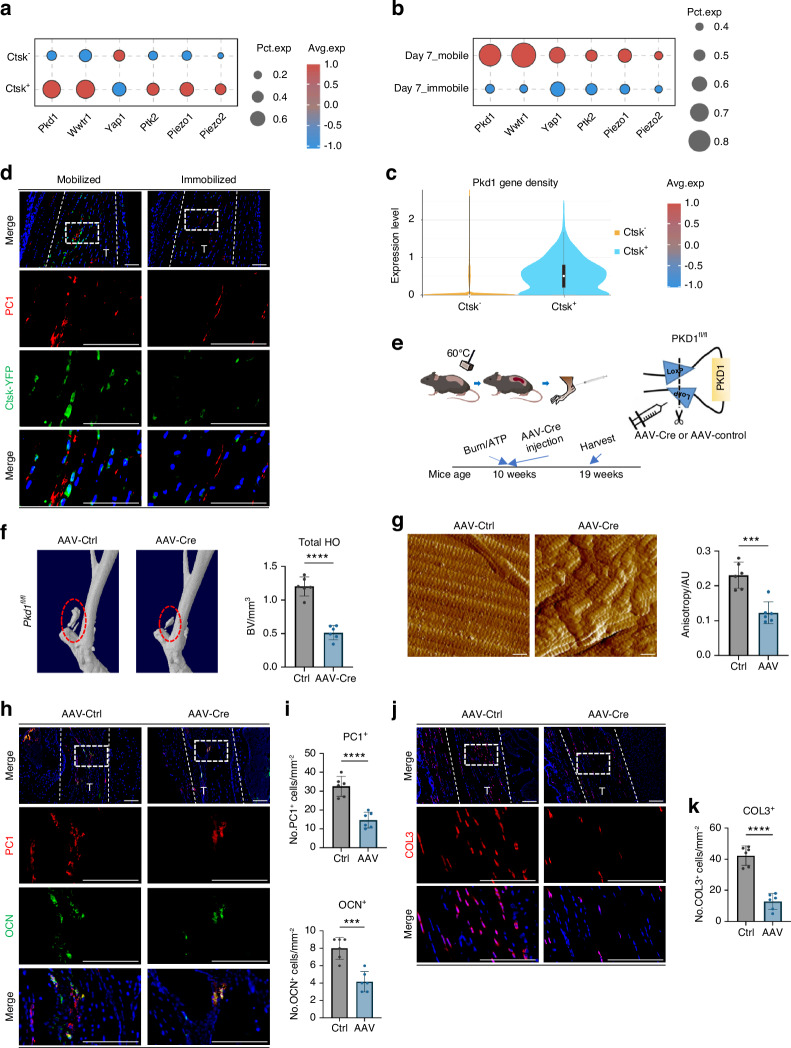
Polycystin-1 conducting mechanical signals affects abnormal osteogenesis and ECM structures after Achilles tendon injury. **a** *Ctsk*^*+*^ cells were isolated from all the cells obtained by single-cell sequencing, and the expression levels of some common mechanosensitive protein genes were analyzed, including *Yap1* (YAP), *Wwtr1* (TAZ), *Ptk2* (FAK), *Pkd1* (PC1), *Piezo1* and *Piezo2*. **b** *Ctsk*^*+*^ TDMSCs were isolated from all the cells obtained by single-cell sequencing, and compared for the effects of mobile or immobile interventions after HO modeling on mechanoreception-related genes expressed in these cells, including *Pkd1*, *Wwtr1*, *Yap1*, *Ptk2*, *Piezo1* and *Piezo2*. **c** Violin plot of *Pkd1* gene expression level in *Ctsk*^*+*^ and *Ctsk*^*-*^ cells. **d** Representative images of immunofluorescence staining with anti-GFP and anti-PC1 of the specimens of Ctsk-YFP lineage-traced mice divided into mobile and fixed groups after Burn/ATP modeling. Cell nuclei were stained with DAPI. The Achilles tendon images at the HO site (top) were marked by white dashed lines at x20 magnification, and the images at x63 magnification (bottom) in the white dashed boxes were selected for statistical analysis of cell counting, showing the antibody staining channels separately. **e** Schematic diagram of the experiment to induce local *Pkd1* knockout by AAV-Cre injection. **f** Representative 3D reconstruction images of MicroCT scanning of the specimens of *Pkd1*^*fl/fl*^ mice injected with AAV-Ctrl and AAV-Cre (2× 10^10^ plaque-forming units per mouse) after Burn/ATP modeling, and statistical analysis results of the bone volume of ectopic ossification shown by MicroCT scanning. **g** Atomic force microscopy scanning images and Anisotropy analysis of the ECM arrangement of the specimens of AAV-Ctrl and AAV-Cre mice after Burn/ATP modeling. Scale bars: 1 μm. **h** Representative images of immunofluorescence staining with anti-PC1 and anti-OCN of the HO site of the specimens of AAV-Ctrl and AAV-Cre mice after Burn/ATP modeling. Scale bars: 100 μm. **i** Statistical comparison of the number of PC1^+^ cells and OCN^+^ cells in the field of view of the images, comparing the Ctrl group and the AAV-Cre injection group by independent samples *t*-test (*n* = 6, ***P < 0.001, ****P < 0.000 1). **j** Representative images of immunofluorescence staining with anti-COL3 of the Achilles tendon site of the specimens of AAV-Ctrl and AAV-Cre mice after Burn/ATP modeling. Scale bars: 100 μm. **k** Statistical comparison of the number of COL3^+^ cells in the field of view of the images, comparing the Ctrl group and the AAV-Cre injection group by independent samples *t*-test (*n* = 6, ****P < 0.000 1)

To further explore how CTSK^+^ cells sense mechanical signal transduction, we analyzed the expression levels of mechanical signal transduction related genes in CTSK^+^ cells via scRNA-seq analysis. Many types of mechanical signal transduction genes were expressed in CTSK^+^ cells. Among several common mechanical signal transduction genes, we found that the *Pkd1* (encoding gene for polycystin-l (PC1)) and *Wwtr1* (coding gene for TAZ) genes presented relatively high expression levels (Fig. 3a). In addtion, during immoblization, the expression of *Pkd1* and *Wwtr1* dramatically decreased (Fig. 3b). Moreover, *Pkd1*^*+*^ cells exhibited enhanced expression of osteogenic and fibrogenic genes. Additionally, the osteogenic or fibrogenic differentiation bias of *Pkd1*^*+*^ cells was observed to vary with different mechanical intervention conditions (Fig. S2E). Given that the PC1-TAZ axis has been shown to regulate bone formation and BMSC differentiation during skeletal modeling and remodeling,^16^ we hypothesized that CTSK^+^ cells mainly sense mechanical stimulation and affect HO through PC1.

To further verify the role of the PC1 protein in local mechanical signal transduction after Achilles tendon injury in vivo, we performed immobilization interventions on a CTSK lineage tracing mouse model. Immunofluorescence staining revealed that in the mobile group, most CTSK-lineage cells were costianed with PC1 in the cell membrane, which significantly decreased with immobilizaiton (Fig. 3d, Fig. S1I). To test the function of PC1 in HO, we injected the AAV-Cre virus into the Achilles tendon of *Pkd1*^*fl/fl*^ mice after Burn/ATP Achilles tendon injury (Fig. 3e). After the virus was injected, *Cre* gene expression was induced in local cells of injured Achilles tendon, thereby achieving local inducible gene knockout in *Pkd1*^*fl/fl*^ mice. Compared with that in the control group, the amount of ectopic ossification decreased with AAV-Cre treatment (Fig. 3f), and the structure of the ECM thus became disorganized(Fig. 3g). Immunofluorescence staining of tissue sections revealed that PC1 protein and OCN^+^ osteoblast expression was reduced after AAV-Cre was injected into *Pkd1*^*fl/fl*^ mice (Fig. 3h, i). Compared with those in the control group, the number of COL3^+^ fibroblasts in the injured area was decreased in the AAV-Cre-injected group (Fig. 3j, k).

### Conditional knockdown of Pkd1 in TDMSCs alters aberrant osteogenesis and the ECM in vivo

**Fig. 4:**
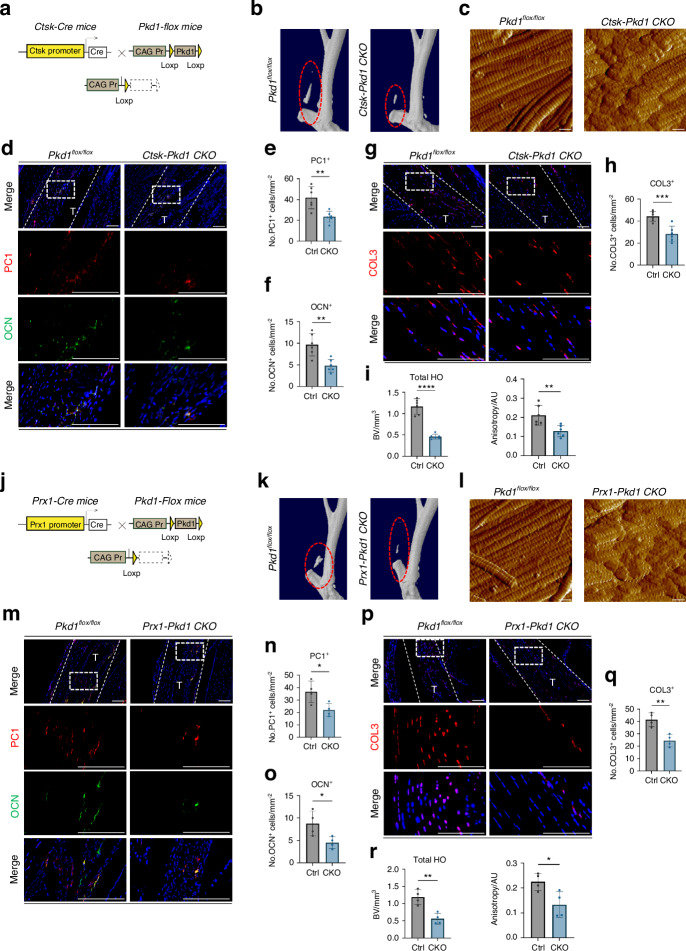
Conditional knockdown of *Pkd1* in TDMSCs alters aberrant osteogenesis and ECM in vivo. **a** Schematic diagram of the conditional gene knockout mouse with *Ctsk*^*+*^ cell-specific knockout of *Pkd1*. **b** Representative 3D reconstruction images of MicroCT scanning of the specimens of *CtskCre*^*−/−*^*Pkd1*^*fl/fl*^ control mice and *CtskCre*^*+/−*^*Pkd1*^*fl/fl*^ knockout mice after Burn/ATP modeling. **c** Atomic force microscopy scanning images of the ECM arrangement of the specimens of *CtskCre*^*−/−*^*Pkd1*^*fl/fl*^ control mice and *CtskCre*^*+/−*^*Pkd1*^*fl/fl*^ knockout mice after Burn/ATP modeling. Scale bars: 1 μm. **d** Representative images of immunofluorescence staining with anti-PC1 and anti-OCN of the HO site of the specimens of *CtskCre*^*−/−*^*Pkd1*^*fl/fl*^ control mice and *CtskCre*^*+/−*^*Pkd1*^*fl/fl*^ knockout mice after Burn/ATP modeling. Cell nuclei were stained with DAPI. Scale bars: 100 μm. **e**, **f** Statistical comparison of the number of PC1^+^ cells and OCN^+^ cells in the field of view of the images, comparing *CtskCre*^*−/−*^*Pkd1*^*fl/fl*^ control mice and *CtskCre*^*+/−*^*Pkd1*^*fl/fl*^ knockout mice by independent samples *t*-test (*n* = 6, **P < 0.01). **g** Representative images of immunofluorescence staining with anti-COL3 of the Achilles tendon site of the specimens of *CtskCre*^*−/−*^*Pkd1*^*fl/fl*^ control mice and *CtskCre*^*+/−*^*Pkd1*^*fl/fl*^ knockout mice after Burn/ATP modeling. Scale bars: 100 μm. **h** Statistical comparison of the number of COL3^+^ cells in the field of view of the images, comparing *CtskCre*^*−/−*^*Pkd1*^*fl/fl*^ control mice and *CtskCre*^*+/−*^*Pkd1*^*fl/fl*^ knockout mice by independent samples *t*-test (*n* = 6, ***P < 0.001). **i** Statistical analysis results of the bone volume of ectopic ossification shown by MicroCT scanning, analyzing total HO Bone Volume, and Anisotropy analysis of AFM images. **j** Schematic diagram of the conditional gene knockout mouse with *Prx1*^*+*^ cell-specific knockout of *Pkd1*. **k** Representative 3D reconstruction images of MicroCT scanning of the specimens of *Prx1Cre*^*−/−*^*Pkd1*^*fl/fl*^ control mice and *Prx1Cre*^*+/−*^*Pkd1*^*fl/fl*^ knockout mice after Burn/ATP modeling. **l** Atomic force microscopy scanning images of the ECM arrangement of the specimens of *Prx1Cre*^*−/−*^*Pkd1*^*fl/fl*^ control mice and *Prx1Cre*^*+/−*^*Pkd1*^*fl/fl*^ knockout mice after Burn/ATP modeling. Scale bars: 1 μm. **m** Representative images of immunofluorescence staining with anti-PC1 and anti-OCN of the HO site of the specimens of *Prx1Cre*^*−/−*^*Pkd1*^*fl/fl*^ control mice and *Prx1Cre*^*+/−*^*Pkd1*^*fl/fl*^ knockout mice after Burn/ATP modeling. Scale bars: 100 μm. **n**, **o** Statistical comparison of the number of PC1^+^ cells and OCN^+^ cells in the field of view of the images, comparing *Prx1Cre*^*−/−*^*Pkd1*^*fl/fl*^ control mice and *Prx1Cre*^*+/−*^*Pkd1*^*fl/fl*^ knockout mice by independent samples *t*-test (*n* = 4, *P < 0.05). **p** Representative images of immunofluorescence staining with anti-COL3 of the Achilles tendon site of the specimens of *Prx1Cre*^*−/−*^*Pkd1*^*fl/fl*^ control mice and *Prx1Cre*^*+/−*^*Pkd1*^*fl/fl*^ knockout mice after Burn/ATP modeling. Scale bars: 100 μm. **q** Statistical comparison of the number of COL3^+^ cells in the field of view of the images, comparing *Prx1Cre*^*−/−*^*Pkd1*^*fl/fl*^ control mice and *Prx1Cre*^*+/−*^*Pkd1*^*fl/fl*^ knockout mice by independent samples *t*-test (*n* = 4, **P < 0.01). **r** Statistical analysis results of the bone volume of ectopic ossification shown by MicroCT scanning, analyzing total HO Bone Volume, and Anisotropy analysis of AFM images

The use of the AAV-Cre virus for inducible gene knockout results in the knockout of *Pkd1* in all local cells. To avoid any confounding effects, we created a CTSK^+^ cell conditional *Pkd1* gene-specific knockout mouse model, *Ctsk-Cre*^*+/−*^*-Pkd1*^*fl/fl*^ CKO mice, to confirm the role of the PC1 protein in CTSK^+^ cells in vivo. *Pkd1*^*fl/fl*^ litters served as control (Fig. 4a). We established the same Burn/ATP injury model in WT and CKO mice. After the specific knockout of the *Pkd1* gene in CTSK^+^ cells, HO formation was significantly reduced (Fig. 4b, i). The arrangement of the ECM was also disorganized (Fig. 4c, i). In addition, the number of osteoblast-like cells and newly formed fibroblast-like cells at the injury site was decreased in CKO mice (Fig. 4d, f, g).

We next generated *Prrx1-Cre*^*+/−*^*-Pkd1*^*fl/fl*^ conditional gene knockout mice (Fig. 4j), to confirm the effect of PC1 on TDMSC mechanical-signal transduction in vivo. The *Prrx1* gene encodes a paired-related homeobox 1 (PRRX1) protein, which is a transcription factor expressed in multiple tissues, especially in neural crest and limb buds, and is generally considered a classical mesenchymal stem cell marker.^20^ After the same Burn/ATP injury model was established in *Prrx1-Cre*^*+/−*^*-Pkd1*^*fl/fl*^ and *Prrx1-Cre*^*−/−*^*-Pkd1*^*fl/fl*^ mice, micro-CT scanning was performed to measure the amount of ectopic ossification. We found that the HO volume in the CKO group was significantly lower than that in the WT group (Fig. 4k, r). *Pkd1* gene knockout in PRRX1^+^ cells impaired mesenchymal cell osteogenic and fibrogenic differentiation (Fig. 4m–q), and resulted in decreased anisotropy and a poor ultrastructure of the ECM (Fig. 4l, r). These data demonstrate that PC1 regulates aberrant TDMSC osteogenic and fibrogenic fates and ECM organization.

### Polycystin 1 transmits mechanical signals through downstream TAZ

**Fig. 5:**
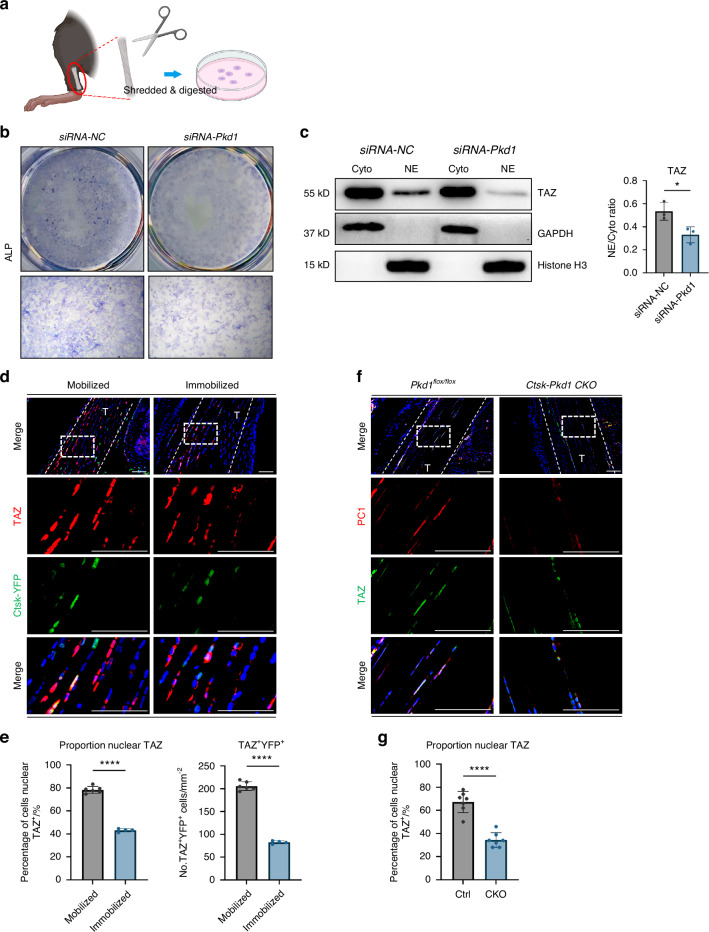
Polycystin 1 transmits mechanical signals through downstream TAZ in Tendon-derived MSCs. **a** Schematic diagram of the extraction of MSCs at the Achilles tendon site. **b** Representative images of ALP staining of Achilles tendon mesenchymal cells after PKD1 siRNA interference, compared with NC siRNA. **c** Separation of cytoplasmic and nuclear proteins of TDMSCs after PKD1 siRNA interference to detect the nuclear translocation of TAZ, compared with Si NC group. Biological repetitions = 3. **d** Representative images of immunofluorescence staining with anti-GFP and anti-TAZ of the specimens of Ctsk-YFP lineage-traced mice divided into mobile and immobile groups after Burn/ATP modeling. Cell nuclei were stained with DAPI. Scale bars: 100 μm. **e** Statistical comparison of the number of TAZ^+^ cells and TAZ nuclear translocation in the field of view of the images, comparing the mobile and fixed groups by independent samples *t*-test (mobile group *n* = 6, fixed group *n* = 4, ***P < 0.001). **f** Representative images of immunofluorescence staining with anti-PC1 and anti-TAZ of the HO site of the specimens of *CtskCre*^*−/−*^*Pkd1*^*fl/fl*^ control mice and *CtskCre*^*+/−*^*Pkd1*^*fl/fl*^ knockout mice after Burn/ATP modeling. Scale bars: 100 μm. **g** Statistical comparison of the number of TAZ nuclear translocation cells in the field of view of the images, comparing *CtskCre*^*−/−*^*Pkd1*^*fl/fl*^ control mice and *CtskCre*^*+/−*^*Pkd1*^*fl/fl*^ knockout mice by independent samples *t*-test (*n* = 6, **P < 0.01)

Previous studies have shown that PC-1 transduces mechanical signals through the downstream transcriptional coactivator TAZ.^15,16^ Earlier studies revealed that TAZ plays an important role in promoting the differentiation of MSCs into osteoblasts and inhibiting their differentiation into adipocytes. TAZ can bind to *Runx2* to form a TAZ-Runx2 complex, which in turn transcriptionally activates osteogenic genes, such as *Osterix* and *Osteocalcin*.^21^ Single-cell analysis data also revealed elevated expression of osteogenic and fibrogenic genes in *Taz*^+^ cells. Moreover, the propensity of *Taz*^+^ cells to differentiate into either osteoblasts or fibroblasts was found to be modulated by varying mechanical stimuli. We then tested whether the PC1-TAZ axis also influences cell functions in a HO mouse model. We isolated TDMSCs from murine Achilles tendons following previous methods,^22–24^ and verified the function of PC1 in vitro (Fig. 5a). The osteogenic activity of tendon mesenchymal stem cells, as measured by ALP staining, decreased after *Pkd1* was silenced with siRNA(Fig. 5b). TAZ nuclear translocation, which mediates downstream signaling, decreased after *Pkd1* was knocked down in contrast to the control group (Fig. 5c). These findings indicate that PC1 may regulate mechanical signal transduction and osteogenic potential in tendon-derived mesenchymal stem cells through TAZ nuclear translocation.

To further investigate the role of the PC1-TAZ mechanical signal transduction pathway in the complex environment of tendon injury in vivo, we generated Burn/ATP injury models in a CTSK-YFP lineage tracing mouse model and divided them into mobile and immobile groups. The results of immunofluorescence staining indicated that CTSK-lineage cells colocalized with TAZ at the injury site. Interestingly, the fluorescence signal of TAZ was more concentrated in the cytoplasm in the immobilized group than in the mobilized control group (Fig. 5d, e), indicating that immobilization suppresses TAZ nuclear translocation. More importantly, the nuclear localization of TAZ was also lower in *Ctsk-Cre*^*+/−*^*-Pkd1*^*fl/fl*^ CKO mice than in *Pkd1*^*fl/fl*^ littermate controls (Fig. 5f, g).

**Fig. 6:**
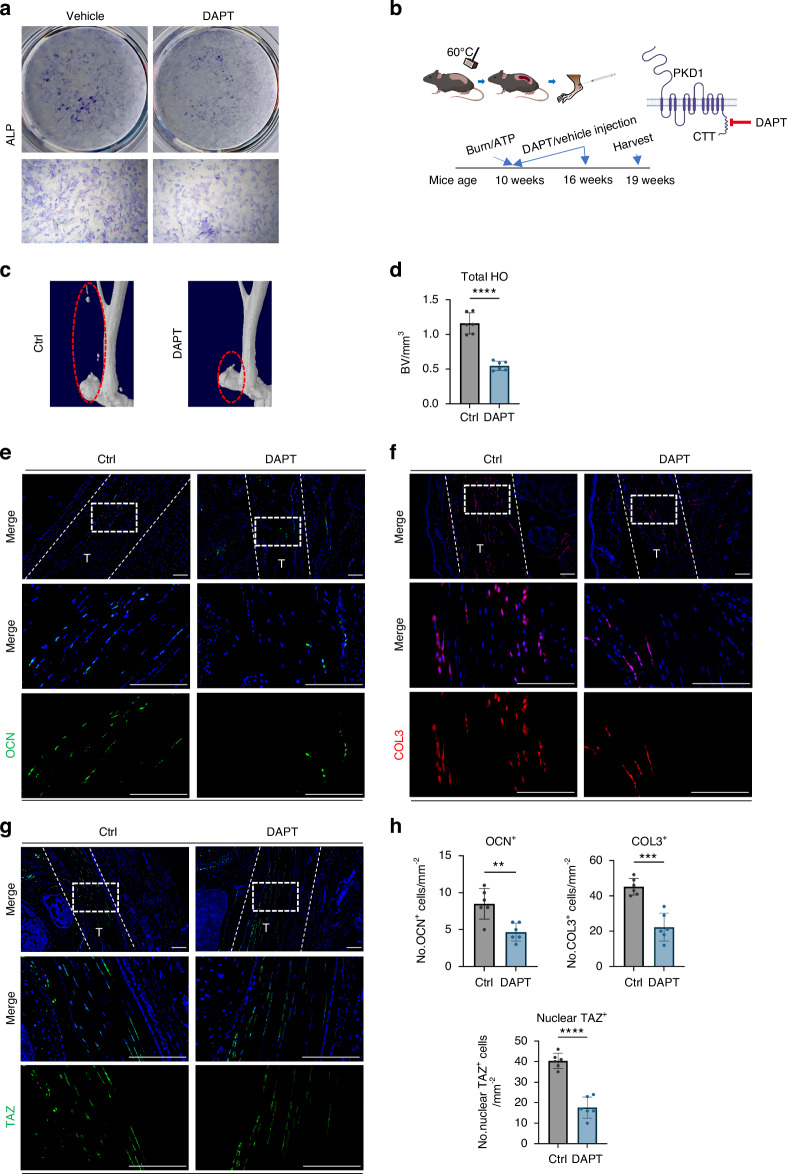
Injection of PC1-CTT inhibitor DAPT attenuates osteogenic and fibrogenic activity after injury. **a** Representative images of ALP staining of Achilles tendon mesenchymal stem cells after DAPT intervention, compared with Vehicle. **b** Schematic diagram of the mouse injected with DAPT after injury modeling. **c** Representative 3D reconstruction images of MicroCT scanning of the specimens of WT mice injected with Vehicle or DAPT (10 mg/kg) after Burn/ATP modeling. **d** Statistical analysis results of the bone volume of ectopic ossification shown by MicroCT scanning. **e** Representative images of immunofluorescence staining with anti-OCN of the HO site of the specimens of Ctrl mice injected with Vehicle and mice injected with DAPT after Burn/ATP modeling. Cell nuclei were stained with DAPI. Scale bars: 100 μm. **f** Representative images of immunofluorescence staining with anti-COL3 of the Achilles tendon site of the specimens of Ctrl mice injected with Vehicle and mice injected with DAPT after Burn/ATP modeling. Scale bars: 100 μm. **g** Representative images of immunofluorescence staining with anti-TAZ of the Achilles tendon site of the specimens of Ctrl mice injected with Vehicle and mice injected with DAPT after Burn/ATP modeling. Scale bars: 100 μm. **h** Statistical comparison of the number of OCN^+^, Nuclear TAZ^+^, and COL3^+^ cells in the field of view of the images, comparing Ctrl mice injected with Vehicle and mice injected with DAPT by independent samples *t*-test (*n* = 6, **P < 0.01, ***P < 0.001, ****P < 0.000 1)

To further confirm the role of the PC1-TAZ pathway in HO and the ECM after tendon injury, DAPT, a γ-secretase inhibitor that inhibits the cleavage of the PC1-CTT terminal and thus inactivates the PC1-TAZ signaling pathway, was used in subsequent experiments.^16,25^ DAPT treatment reduced the osteogenic activity of tendon-derived mesenchymal stem cells in vitro, as evidenced by decreased ALP activity (Fig. 6a). Additionally, DAPT may also simultaneously affect the Notch pathway. To investigate through which pathway DAPT mainly exerts its influence, we further compared the inhibitory effect of DAPT on TDMSCs with that of IMR-1, a specific inhibitor of the Notch pathway. After IMR-1 inhibited the Notch pathway, DAPT had a further inhibitory effect, suggesting that DAPT v has targets other than the Notch pathway. Moreover, after we overexpressed membrane-bound PC1-CTT, its overexpression was inhibited by DAPT. This finding also indicates, to some extent, that DAPT does play a role by affecting PC1-CTT. We then tested the effect of DAPT in vivo by local subcutaneous injection of DAPT around the tendon injury site in Burn/ATP HO model mice treated with 10 mg/kg DAPT twice a week (Fig. 6b). Compared with vehicle treatment, DAPT treatment dramatically suppressed HO formation (Fig. 6c, d), and reduced the number of osteoblast-like cells and fibroblast-like cells in injured tendons (Fig. 6e, f). As expected, the localization of TAZ to the nucleus was inhibited by DAPT administration (Fig. 6g). These data suggest that PC1-CTT cleavage-induced TAZ nuclear translocation promotes the osteogenic and fibrogenic differentiation of MSCs and HO progression.

### Conditional knockdown of Taz in TDMSCs alters aberrant osteogenesis and the ECM in vivo

**Fig. 7:**
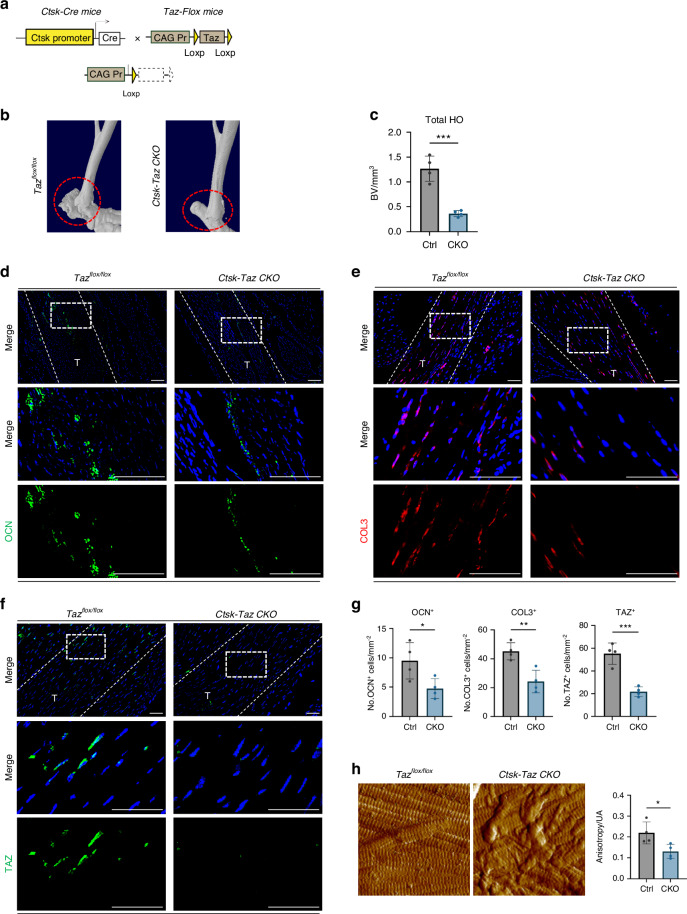
Conditional knockdown of *Taz* in CTSK^+^ TDMSCs alters aberrant osteogenesis and ECM in vivo. **a** Schematic diagram of the conditional gene knockout mouse with *Ctsk*^*+*^ cell-specific knockout of *Taz*. **b** Representative 3D reconstruction images of MicroCT scanning of the specimens of *CtskCre*^*−/−*^*Taz*^*fl/fl*^ control mice and *CtskCre*^*+/−*^*Taz*^*fl/fl*^ knockout mice after Burn/ATP modeling. **c** Statistical analysis results of the bone volume of ectopic ossification shown by MicroCT scanning, analyzing total HO Bone Volume. **d** Representative images of immunofluorescence staining with anti-OCN of the HO site of the specimens of *CtskCre*^*−/−*^*Taz*^*fl/fl*^ control mice and *CtskCre*^*+/−*^*Taz*^*fl/fl*^ knockout mice after Burn/ATP modeling. Cell nuclei were stained with DAPI. Scale bars: 100 μm. **e** Representative images of immunofluorescence staining with anti-COL3 of the Achilles tendon site of the specimens of *CtskCre*^*−/−*^*Taz*^*fl/fl*^ control mice and *CtskCre*^*+/−*^*Taz*^*fl/fl*^ knockout mice after Burn/ATP modeling. **f** Representative images of immunofluorescence staining with anti-TAZ of the Achilles tendon site of the specimens of *CtskCre*^*−/−*^*Taz*^*fl/fl*^ control mice and *CtskCre*^*+/−*^*Taz*^*fl/fl*^ knockout mice after Burn/ATP modeling. **g** Statistical comparison of the number of OCN^+^, TAZ^+^, and COL3^+^ cells in the field of view of the images, comparing *CtskCre*^*−/−*^*Taz*^*fl/fl*^ control mice and *CtskCre*^*+/−*^*Taz*^*fl/fl*^ knockout mice by independent samples *t*-test (*n* = 4, *P < 0.05, **P < 0.01, ***P < 0.001). **h** Atomic force microscopy scanning images of the ECM arrangement of the specimens of *CtskCre*^*−/−*^*Taz*^*fl/fl*^ control mice and *CtskCre*^*+/−*^*Taz*^*fl/fl*^ knockout mice after Burn/ATP modeling. Scale bars: 1 μm

To further verify the role of TAZ in the mechanical signal transduction pathway in CTSK^+^ cells after tendon injury, we crossed *Ctsk-Cre*^*+/−*^ mice with *Taz*^*fl/fl*^ mice to generate CTSK^+^ cell conditional *Taz* gene specific knockout mice, followed by Achilles tendon injury *Taz*^*fl/fl*^ litters served as controls (Fig. 7a). First, the HO phenotype was analyzed. Compared with *Taz*^*fl/fl*^ mice, HO formation at both the proximal and distal sites was significantly lower in *Ctsk-Cre*^*+/−*^*-Taz*^*fl/fl*^ mice (Fig. 7b, c). Immunofluorescence staining analysis revealed that both osteoblast-like cells and fibroblast-likes cells were diminished in *Ctsk-Cre*^*+/−*^*-Taz*^*fl/fl*^ mice (Fig. 7d, e). We verified the depletion of *Taz* in CTSK^+^ cells by detecting the TAZ fluorescence signal and detected a significantly lower TAZ signal in situ (Fig. 7f). Moreover, ECM alignment became disorganized with TAZ depletion (Fig. 7h). These data indicate that TAZ in CTSK^+^ cells is required for HO formation and ECM organization and that blocking TAZ is sufficient to prevent or alleviate HO.

**Fig. 8:**
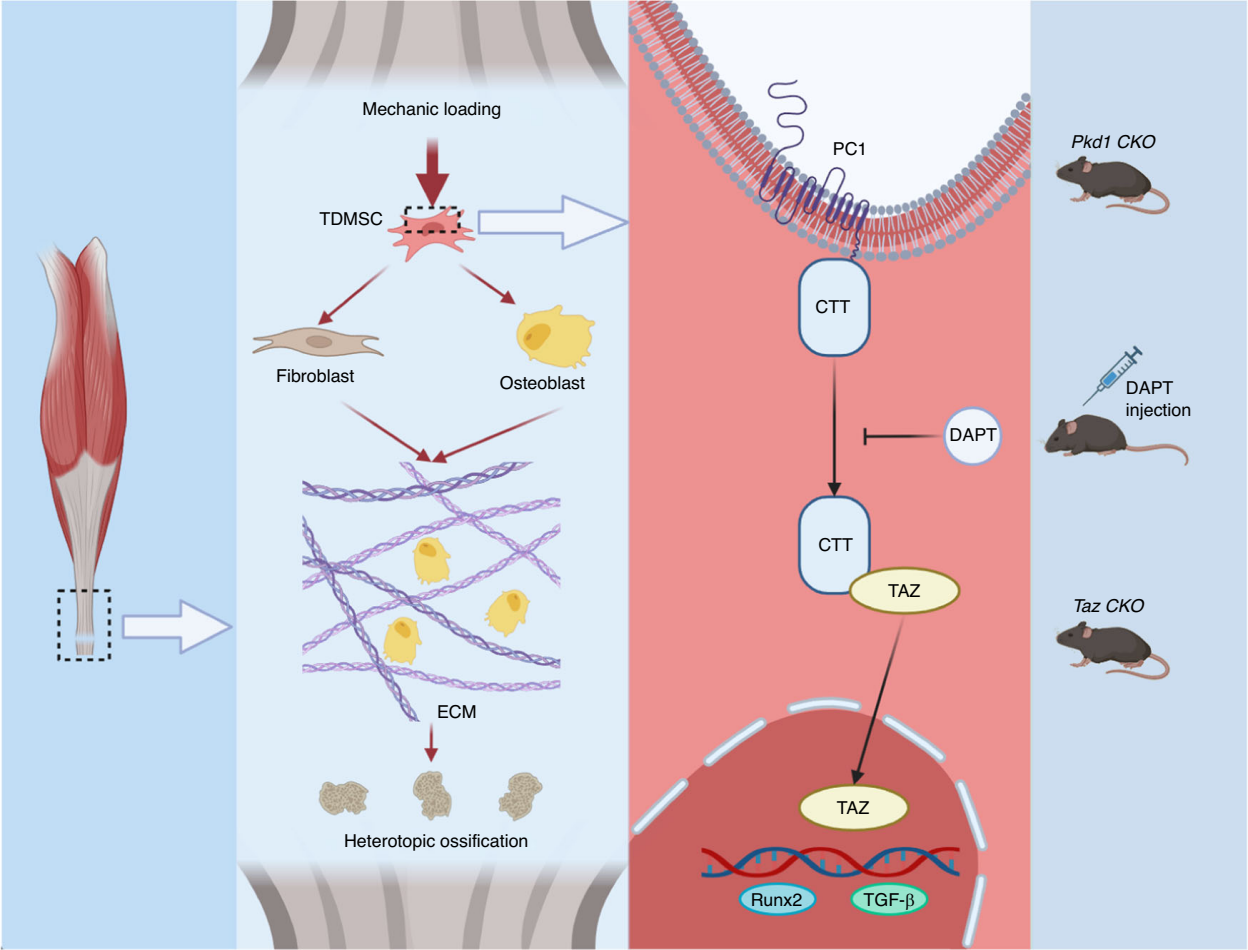
Schematic representation of TDMSCs sensing mechanical signals through the PC1-TAZ axis thus osteogenic and fibroblastic differentiation affecting ECM and HO. Mechanical force promotes osteogenesis and fibrogenesis through PC1-CTT and TAZ nuclear translocation in tendon-derived mesenchymal stem cells

These results indicate that mechanical force stimulates osteogenesis and fibrogenesis, thereby influencing heterotopic ossification and matrix organization, via PC1-CTT and TAZ nuclear translocation in tendon-derived mesenchymal stem cells (Fig. 8).

## Discussions

Mechanical stress modulates bone formation and ECM organization. However, the mechanically sensitive cell populations in HO and the underlying mechanism remain elusive. Our study demonstrated the pivotal role of PC1 in regulating CTSK-lineage tendon-derived mesenchymal stem cells fate and ECM organization, as well as heterotopic ossification in mice. Conditional gene depletion of *Pkd1* or *Taz* in CTSK^+^ cells and pharmaceutical intervention of PC1-TAZ axis attenuate HO progression. Currently, there is no effective drug or treatment for heterotopic ossification and ECM disorder after injury. This study implies a novel strategy for preventing HO by targeting PC1-mediated mechanotransduction.

Previous research revealed various cell origins of heterotopic bone formation, such as endon (Scx-Cre),^26^ endoneurium (Wnt1-CreERT),^27^ interstitial/perivascular cells (Gli1-Cre),^28^ mesenchymal progenitor cells (Prx1-Cre; Dermo1-Cre; Ap2-Cre)^5^ and others. An earlier study has discovered that CTSK labels a subset of Tendon progenitor cells with augmented stemness and osteogenic potential. Deletion of *Sufu* in CTSK^+^ cells activates *Gli1* promoter and elicits Hh pathway target gene expression, resulting in spontaneous ectopic bone formation.^13^ However, which type of MSCs sense mechanical stress, thus affecting cell fate in HO is not clear. In this study, we confirmed that CTSK-lineage tendon-derived mesenchymal stem cells committed into osteogenic and fibrogenic aberrant fate in injury-induced HO. Fibroblasts are the main cells accountable for ECM synthesis.^29,30^ It is widely accepted that collagen, the most prominent substance in the ECM, is secreted by various fibroblasts and regulates its assembly.^31,32^ Moreover, poor ECM quality weakens tissue.^33^ Previous studies also found that the properties of the ECM strongly influenced the osteogenic activity of cells.^34^ Furthermore, immobilization affected the arrangement of the ECM and thus altered the fate differentiation of MSCs; after immobilization, the arrangement of the ECM became more disorganized, and the MSCs were more inclined toward lipogenesis than osteogenesis.^4^ For the first time, our study shows that CTSK^+^ cells are involved in traumatic HO formation not only through their direct osteogenesis after differentiation, but also by modifying the organization of the ECM. Additionally, manipulating CTSK^+^ cells at the gene level to impair their mechanotransduction affects heterotopic ossification and ECM. These findings indicate that CTSK-lineage tendon-derived mesenchymal stem cells conttribute to the abnormal ossification process by differentiating into osteoblasts- and fibroblast-like cells, thus affecting ossification and ECM organization.

We revealed that CTSK-lineage tendon-derived mesenchymal stem cells contributed to heterotopic ossification and ECM in a mechanosensitive manner. Immobilization reduced mechanical stress and diminished heterotopic ossification and fibrosis with attenuated PC1-TAZ signaling. Inhibiting or deleting the PC1-TAZ mechanotransduction pathway decreased ectopic ossification and ECM formation. Cells utilize various mechanisms to sense mechanical forces, such as Piezo1 and Piezo2 for membrane tension,^35–37^ TRPM7, a transport enzyme-coupled ion channel for fluid shear stress,^38^ and Integrin, a transmembrane receptor for extracellular matrix tension.^32^ We showed that CTSK^+^ cells transmitted biomechanical responses through transmembrane mechanosensitive proteins PC1. Previous research indicated that immobilization altered osteogenic and adipogenic differentiation of mesenchymal stem cells via FAK, YAP/TAZ signaling pathway and Discoidin domain receptor 2.^4,9^ Furthermore, mechanotransduction was reported to modulate wound-resident cells,^39^ especially fibroblasts. Increased wound tension upregulated pro-fibrotic genes in fibroblasts, such as collagen, transforming growth factor-β (TGF-β).^40^ Reducing wound tension significantly changed ECM.^41^ Previous studies have shown that the mechanosensory protein TRPV4 on MSC regulated extracellular collagen matrix alignment.^18^ However, the role of PC1-TAZ in either HO formation or fibrogenic cell fate of MSCs is unclear. Our findings corroborate and expand previous findings. Immobilization inhibited HO formation by affecting not only the osteogenic and adipogenic differentiation of PRX1^+^ stem cells but also the involvement of CTSK^+^ stem cells. Moreover, CTSK lineage tendon-derived mesenchymal stem cells played a role in post injury fibrosis. Tracking and locating a mesenchymal cell population that engaged in those post injury pathologies improved our understanding of the disease mechanism and provided a new avenue for intervention and regulation of clinical symptoms.

One limitation of this study is that we focused on acquired HO, also known as traumatic HO, while hereditary HO, known as fibrodysplasia ossificans progressiva (FOP), was not investigated.^42^ Previous studies have suggested some pathological similarities between acquired HO and FOP, but they have distinct pathogenic mechanisms,^43^ and further studies are needed to confirm the effects of PC1-mediated TDMSCs fate and ECM in FOP. Another limitation is that HO has multiple cellular sources, and this study only examined the role of CTSK^+^ TDMSCs, leaving the question of whether other cells involved in HO formation are influenced by mechanical stimulation and affect HO formation unanswered, which is the focus of our future research. Finally, the injection of DAPT inhibits HO, but this treatment needs to be optimized. Frequent injection injuries may induce HO formation, so alternative delivery modes or slow-release systems need to be developed. Moreover, the injected drug may have negative effects on other cells in the local area, so a specific drug delivery system for CTSK^+^ cells needs to be designed to prevent unwanted side effects.

Despite these limitations, our results show that CTSK-lineage TDMSCs are injury-responsive and mechanosensitive cells with the potentials to affect osteogenesis and ECM through the PC1-TAZ axis. We demonstrated that inhibition of PC1 protein and its downstream mechanotransduction pathway on TDMSCs in vivo reduced heterotopic ossification and ECM organization. This suggests the potential pharmaceutical targeting of PC1 for the treatment or prevention of heterotopic ossification. Currently, there is no effective drug or treatment for heterotopic ossification and ECM disorder after injury. Surgery may accelerate HO formation and cause HO recurrence.^44^ This study offers a novel approach for preventing HO by targeting PC1-mediated mechanotransduction in the injured site.

## Methods

### Animal use

All mice were on a C57BL/6 J background and housed in a standard, specific pathogen-free facility at the Central South University Experimental Animal Research Center, with temperature controlled at 22 °C–24 °C and a 12-hour dark/light cycle. They had access to standard food (SJA Experimental Animal Company, China, Hunan) and water, and were provided with environmental enrichment. The mice were healthy and used for internal breeding to generate enough mice for the experiments. Only male mice were used and littermates served as controls. The experiments used 10-week-old mice. All animal care protocols and experiments were reviewed and approved by the Animal Care and Use Committee of Xiangya Medical College Experimental Animal Research Center and the Animal Care and Use Committee of Xiangya Medical College Experimental Animal Research Center, Central South University. The *Pkd1*-floxed mice and *Taz*-floxed mice were constructed using CRISPR/Cas9 technology in GemPharmatech Co., Ltd (China). The flox sequences (ATAACTTCGTATAGCATACATTATACGAAGTTAT) were inserted separately in the two terminals of *Pkd1* exons 2–16 and *Taz* exons 5–10. The *CTSK-Cre* mice were provided by S. Kato (University of Tokyo). The *Prx1*-Cre mice were got from Cyagen Biosciences (China). For genotyping, genomic DNA was extracted from tail tips. The primers of genotyping for mice are: YFP F: AGG GCG AGG AGC TGT TCA; YFP R: TGA AGT CGA TGC CCT TCA G; YFP WT F: CTG GCT TCT GAG GAC CG; YFP WT R: CAG GAC AAC GCC CAC ACA; PKD1-1 F: GAACTTGGCATACGGCATCG; PKD1-1 R: TGGGCAGGCTCTTTATCCAAA; TAZ F: ATGGTGTCTACCAGAAAGGGAT; TAZ R: CGGTGATTTTCAACATGCCACA; Cre-F: AGCGATGGATTTCCGTCTCTGG; Cre-R: AGCTTGCATGATCTCCGGTATTGAA.

### Burn/ATP HO model

The burn/ATP mouse model was performed as described in previous studies.^3,4,45,46^ Ten-week-old male mice were anesthetized with pentobarbital. A 27-gauge needle was inserted percutaneously through the lateral side of the left Achilles tendon, and this procedure was repeated five times at different locations of the tendon. For sham surgery, the needle pierced the skin but did not touch the tendon. Afterwards, the back hair was shaved close to the skin in the ATP or SHAM group, and a 60 °C aluminum block was applied to the back for 18 s, covering an area exceeding 30% of the total body surface area, to induce partial-thickness burn injury. The same surgeon performed the burn/puncture HO model on all animals.

### Joint immobilization

Joint immobilization was performed immediately after injury as described previously.^4,19,47^ Briefly, the immobilizer was made from a 1.5 mL Eppendorf tube. The lid was removed and the edges were smoothed with a blade. The end of the tube was cut off to allow air circulation around the limb. A sponge pad was applied to the proximal end for soft padding to prevent pressure sores. The leg was placed in the immobilizer with the foot in plantar flexion and the knee in extension, and the immobilizer was secured to the body with biocompatible strong glue.

### Micro-CT analysis

The left leg of the mouse was dissected below the knee joint and fixed with 4% paraformaldehyde for 24 h, then scanned and analyzed using high-resolution μCT (Skyscan 1172, Bruker MicroCT, Kontich, Belgium). The parameters of ectopic bone formation at the Achilles tendon injury site were analyzed using NRecon image reconstruction software version 1.6 (Bruker MicroCT), CTAn data analysis software version 1.9 (Bruker MicroCT) and CTVol three-dimensional model visualization software 2.0 (Bruker MicroCT). The scanner was set at 50 kVp, 201 mA, and a resolution of 12.64 mm/pixel. For distal HO attached to the calcaneus, the region of interest (ROI) selected for analysis was separated from the denser calcaneus in the coronal, sagittal, and cross-sectional views, and only ectopic bone was selected to determine the volume of ectopic bone tissue (Tb.BV). The same method was used to select ROI for analyzing proximal HO formed in the soft tissue.

### Immunohistochemical and Immunofluorescence

The mice were carefully dissected from the injured hind limb to obtain HO anlagen after cardiac perfusion and fixed with 4% paraformaldehyde for 24 h at 4 °C. Decalcification was performed in 10% EDTA for 14–21 days. The specimens were cryoprotected in 25% sucrose overnight, embedded in OCT, and sectioned at 7 μm for immunofluorescence staining. Alternatively, they were paraffin-embedded and sectioned at 5 μm for immunohistochemical staining. The sections were blocked with 5% BSA after citrate buffer heat antigen retrieval. The sections were labeled with the following antibodies: GFP(ab290, 1:200, Abcam), OCN(TKRM173, 1:500, TAKARA), PDGFRα(ab203491, 1:500, Abcam), COL3(ab184993, 1:100, Abcam), polycystin-1 antibody (7E12, sc-130554, Santa Cruz), polycystin-1 antibody (C-20, sc-10372, Santa Cruz), TAZ(23306-1-AP, 1:200, proteintach), TAZ(E8E9G, 1:200, Cell Signaling Technology).

The staining specificity of each test sample was assessed by using appropriate primary antibodies and negative controls simultaneously. The antibody-labeled sections were imaged using a fluorescence microscope (Leica N2-DM4B) equipped with DAPI, single-, and dual-cube 488 nm/TRITC filters connected to an Leica DFC7000 T high-resolution digital camera. Each imaging site was imaged in all channels, and the images were only examined and quantified in Leica LAS X or Image J after overlaying in Photoshop. The images were adjusted for brightness and contrast identically between comparison groups. Paraffin sections were deparaffinized or frozen sections were thawed out of OCT and examined by atomic force microscopy (BRUKER, Dimenision ICON). To quantify the orientation and anisotropy of fibrillar structures for downstream analysis, we leveraged the previously validated ImageJ plugin, FibrilTool, which exploits the eigenvectors of the Hessian matrix.^48^ For inter-sample statistical comparisons of anisotropy, we analyzed five regions of interest per digitally scanned biological specimen, following the protocol outlined by the software’s developers. The final data for each sample represent the mean anisotropy values across these designated regions.

### Isolation and characterization of tendon-derived MSCs

We used 6–8-week-old mice for this study. After euthanasia, the Achilles tendons were excised. Tendon-derived MSCs isolation was performed as follows according to previous studies.^22,23^ Briefly, the tendons were thoroughly minced and digested with type I collagenase (3 mg/mL; Sigma) at 37 °C for 1 h, and the suspension was passed through a 70 μm cell filter to generate a single-cell suspension. The cells were cultured in complete medium (α-MEM medium supplemented with 10% fetal bovine serum, 100 U/mL penicillin, and 100 μg/mL streptomycin). After 7–10 days of culture, the cells were detached in 0.1% trypsin solution at 37 °C, the digestion was terminated with complete medium, and then the cell suspension was washed and MSCs were reseeded into culture dishes at low density (400 cells/cm^2^). The medium was changed every 3 days, and the MSCs were passaged when they reached 80% confluence. The fourth passage MSCs were used for all our experiments. All cells were grown at 37 °C in a 5% CO_2_ humid atmosphere.

### Bioinformatics analysis of single-cell RNA-seq

The raw read data were preprocessed. The raw reads (GEO: GSE150995) were processed with fastQC and fastp to remove low-quality reads. Poly-A tails and adapter sequences were removed by cutadapt. After quality control, the reads were mapped to the reference genome Rnor_6.0 using STAR. Gene counts and UMI counts were obtained by featureCounts software. Expression matrix files based on gene counts and UMI counts (Singleron Biotechnologies, Nanjing, China) were generated for subsequent analysis. Quality control, dimensionality reduction, and clustering were then performed. Cells with gene counts lower than 500 were filtered out. Cells with mitochondrial content exceeding 10% were also removed. After filtering, 8 761 cells were retained for downstream analysis. We used functions in Seurat V3.1.2 (Satija et al., 2015) for dimensionality reduction and clustering. All gene expression was normalized and scaled using NormalizeData. FindVariableFeutres selected the top 2 000 variable genes for PCA analysis. The cells were divided into 22 clusters by FindClusters, using the first 20 principal components and a resolution parameter of 1.0. One of the 22 cell types was assigned to each cell cluster by scoring the normalized expression of the following typical markers: neutrophils (Lyz2, Srgn, S100a9, Csf3r), macrophages (Cd68, Csf1r, Lyz2), mesenchymal stem cells (Pdgfra, Prrx1), fibroblasts (Col3a1, Col5a2), myogenic cells (Tnnt3, Mylpf), dendritic cells (Cd74, Irf8), plasma cells (Cd79b, Jchain, Mzb1), B cells (Cd79b, Cd19). We then performed differential gene expression (DEG) analysis. Genes that were expressed in more than 10% of the cells in a cluster and had an average log (fold change) greater than 0.25 were selected as DEGs by Seurat FindMarkers (V3.1.2) based on a Wilcoxon rank-sum test using default parameters. Pathway enrichment analysis was performed using gene ontology (GO) and Kyoto encyclopedia of genes and genomes (KEGG) analysis using the “clusterProfiler” R package version 3.5.1 (Yu et al., 2012). Pathways with a p_adj value less than 0.05 were considered significantly enriched.

### Real-time quantitative RT-PCR and Western blot analysis

For real-time quantitative RT-PCR, 1.0 μg of total RNA isolated from specimens was reverse transcribed as previously outlined. PCR reactions comprised 20 ng of template cDNA, 375 nmol/L of each forward and reverse primer, and 1× EvaGreen Supermix (Bio-Rad) in a 10 μL volume. The threshold cycle (Ct) of the target gene product from the specified genotype was normalized to the Ct for cyclophilin A. Subsequently, the target gene product relative to cyclophilin A was normalized to the mean ratio of the wild-type or control group, arbitrarily set to 1.

For Western blot analysis, supernatant protein concentrations were quantified using a total protein assay kit (Bio-Rad). Equal protein amounts were resolved on 4%–12% Bis-Tris or 3%–8% Tris-Acetate gradient gels (Invitrogen) and subjected to standard Western blot procedures as previously documented. Nuclear protein translocation was assessed by isolating cytoplasmic and nuclear fractions prior to Western blot analysis. At least 3 biological replicates of each assay were performed.

### Quantification and statistical analysis

Data are presented as mean ± standard deviation. Data are continuous and normally distributed. A two-tailed Student’s *t*-test was used to compare two groups. One-way or two-way analysis of variance was used to compare multiple groups. All in vitro experiments were repeated at least three times and representative experiments are shown. Differences were considered significant when *P* < 0.05. The sample sizes for in vivo and in vitro experiments were based on previous experience. All samples were randomly assigned and analyzed in a blinded manner. No initial exclusion criteria were used for in vitro and in vivo experiments, and no animals or replicates were excluded from the study.

## Supplementary information

Supplementary Information

Supplementary Information

## Data Availability

The data that support the findings of this study are available within the article and its supplementary materials or from the corresponding author upon reasonable request.
