# Supplementary material for: Polycystin-1 regulates tendon-derived mesenchymal stem cells fate and matrix organization in heterotopic ossification
Source: Bone Res. 2025 Jan 20;13:11. doi: 10.1038/s41413-024-00392-y (PMC11746979; doi:10.1038/s41413-024-00392-y)
Supplement: Supplementary file 1 — Supplementary Information [file 41413_2024_392_MOESM1_ESM.pdf]

Figure S1

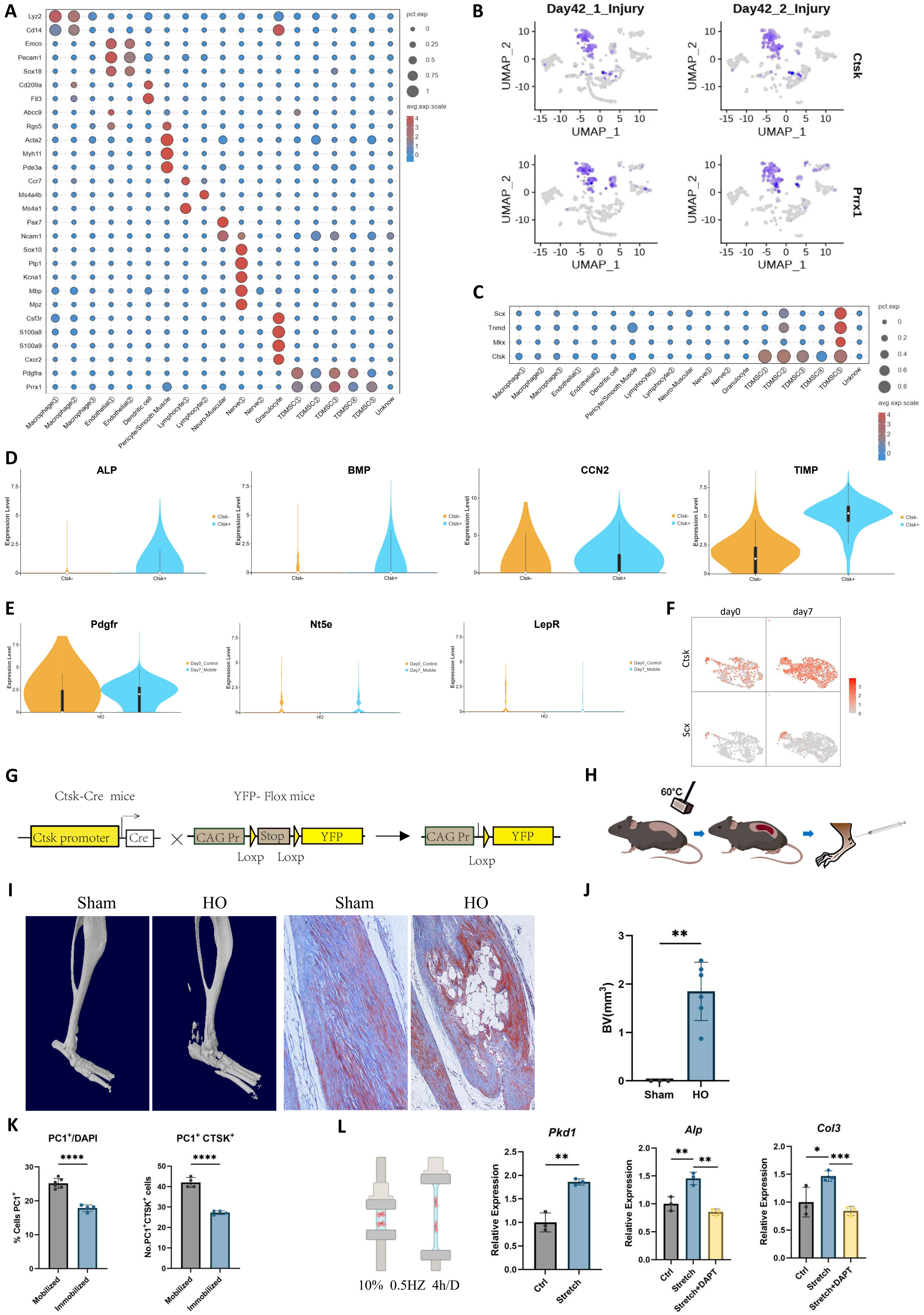
A
B
Day42_1_Injury
Day42_2_Injury
Ctsk
Prrx1
UMAP_1
UMAP_2
C
D
ALP
BMP
CCN2
TIMP
Expression Level
Ctsk-
Ctsk+
E
Pdgfr
Nt5e
LepR
Day0_Control
Day7_Mobile
HO
F
day0
day7
Ctsk
Scx
G
Ctsk-Cre mice
YFP- Flox mice
Ctsk promoter
Cre
CAG Pr
Stop
YFP
Loxp
H
60°C
I
Sham
HO
J
BV(mm³)
Sham
HO
K
PC1⁺/DAPI
PC1⁺ CTSK⁺
% Cells PC1⁺
No.PC1⁺CTSK⁺ cells
Mobilized
Immobilized
L
10% 0.5HZ 4h/D
Pkd1
Alp
Col3
Relative Expression
Ctrl
Stretch
Stretch+DAPT

Figure S2

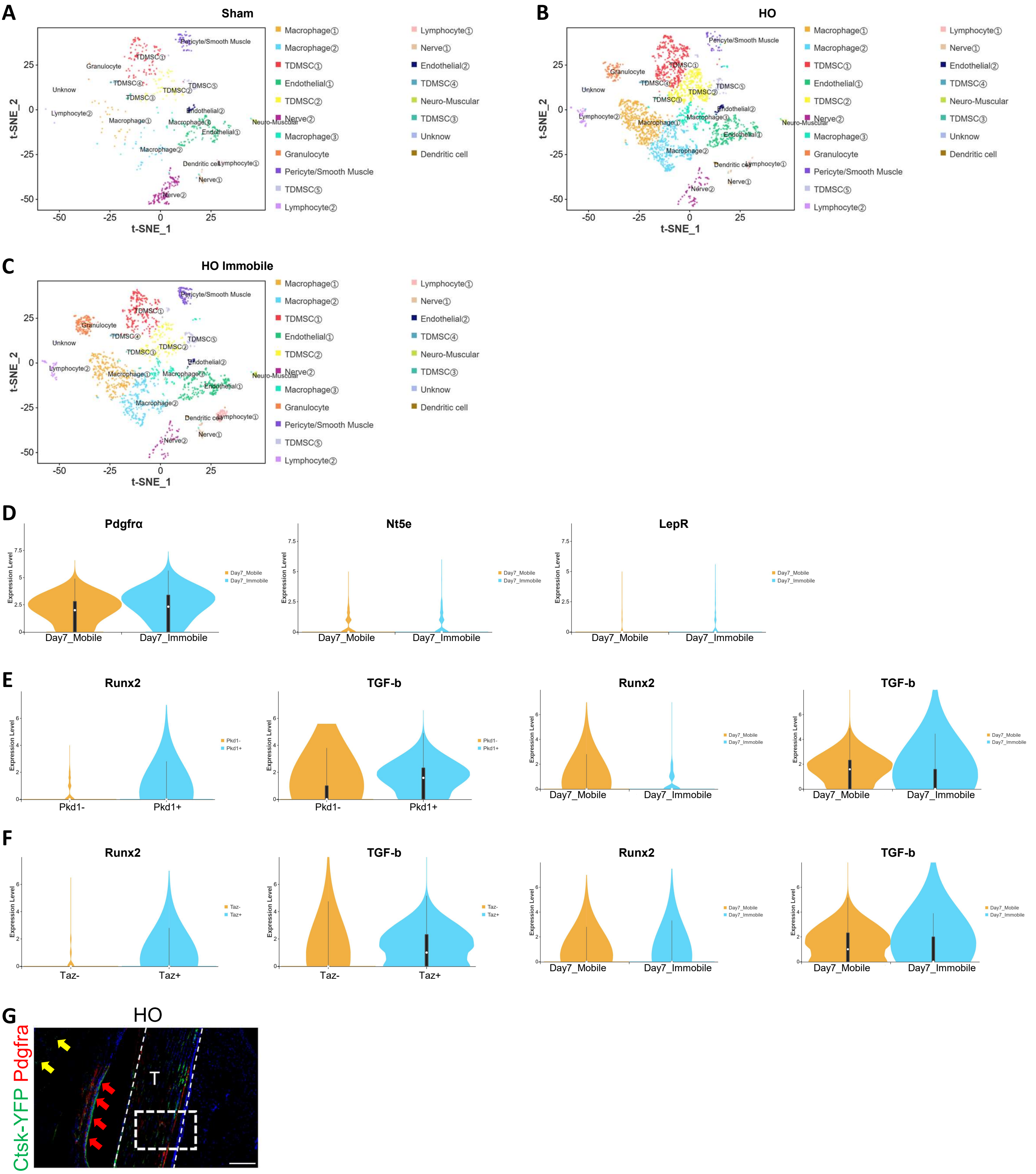

A
Sham
B
HO
C
HO Immobile
t-SNE_1
t-SNE_2
Macrophage①
Macrophage②
TDMSC①
Endothelial①
TDMSC②
Nerve②
Macrophage③
Granulocyte
Pericyte/Smooth Muscle
TDMSC⑤
Lymphocyte②
Lymphocyte①
Nerve①
Endothelial②
TDMSC④
Neuro-Muscular
TDMSC③
Unknow
Dendritic cell
D
Pdgfrα
Nt5e
LepR
Expression Level
Day7_Mobile
Day7_Immobile
E
Runx2
TGF-b
Pkd1-
Pkd1+
F
Taz-
Taz+
G
HO
T
Ctsk-YFP Pdgfra

Figure S3

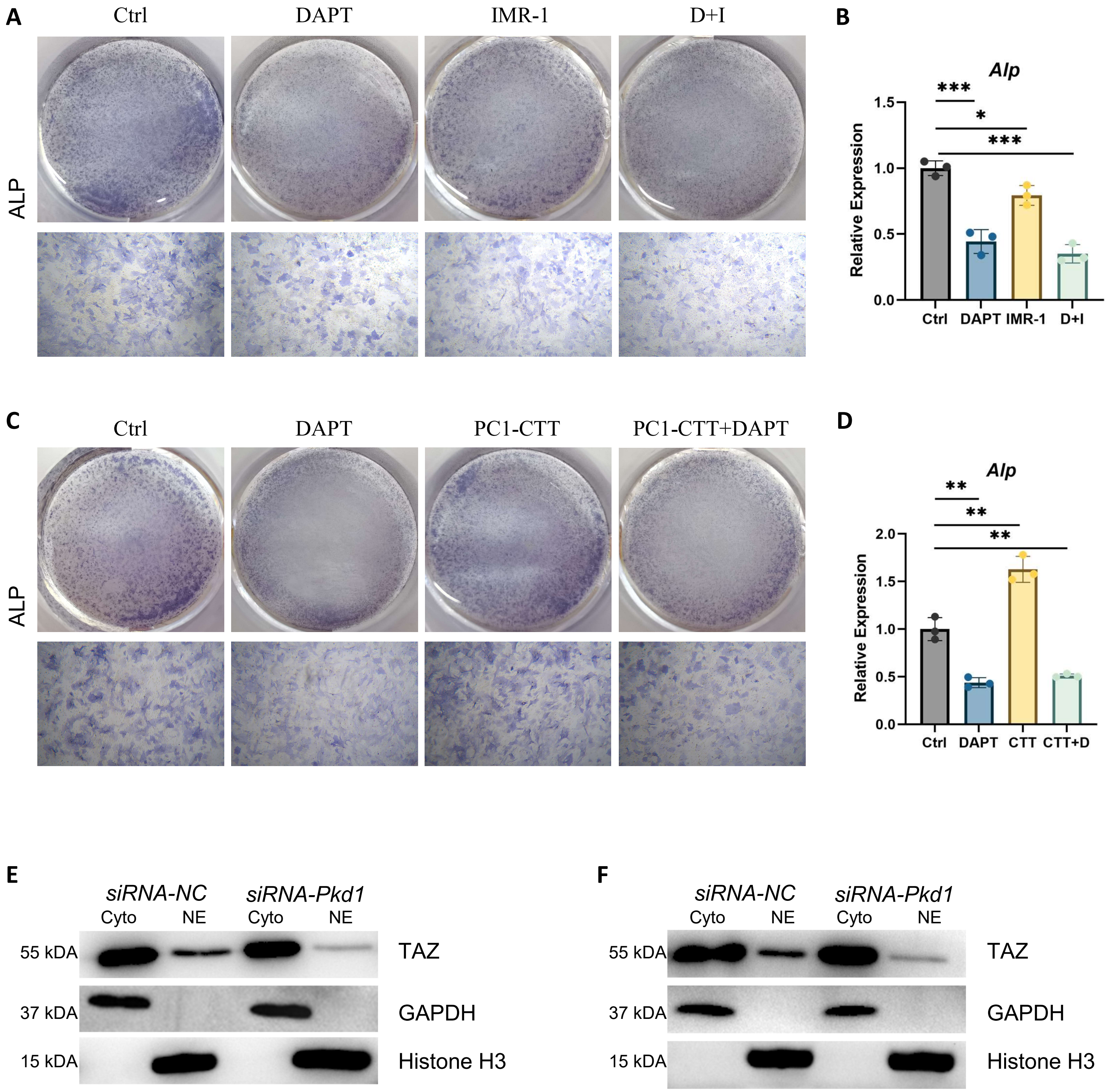
A
Ctrl
DAPT
IMR-1
D+I
ALP
B
Alp
Relative Expression
1.5
1.0
0.5
0.0
***
*
***
Ctrl
DAPT
IMR-1
D+I
C
Ctrl
DAPT
PC1-CTT
PC1-CTT+DAPT
ALP
D
Alp
Relative Expression
2.0
1.5
1.0
0.5
0.0
**
**
**
Ctrl
DAPT
CTT
CTT+D
E
siRNA-NC
siRNA-Pkd1
Cyto
NE
Cyto
NE
55 kDA
37 kDA
15 kDA
TAZ
GAPDH
Histone H3
F
siRNA-NC
siRNA-Pkd1
Cyto
NE
Cyto
NE
55 kDA
37 kDA
15 kDA
TAZ
GAPDH
Histone H3
