# Supplementary material for: Polycystin-1 regulates tendon-derived mesenchymal stem cells fate and matrix organization in heterotopic ossification
Source: Bone Res. 2025 Jan 20;13:11. doi: 10.1038/s41413-024-00392-y (PMC11746979; doi:10.1038/s41413-024-00392-y)
Supplement: Supplementary file 2 — Supplementary Information [file 41413_2024_392_MOESM2_ESM.docx]

**Supplemental Figure 1**. (A) Expression levels and percentages of marker genes after cell clustering. (B) Analysis of single-cell data on the distribution of *Ctsk+* as well as *Prrx1+* genes during the late stage of injury. (C) Comparison of the distribution of *Ctsk* genes with tendon-specific genes *Scx*, *Tnmd*, and *Mkx* in single-cell data analysis. (D) Comparison of the expression of osteogenesis-related genes *Alp* and *Bmp* and fibrogenesis-related genes *Ccn2* and *Timp* in *Ctsk+* and *Ctsk-* cells in single-cell sequencing data. (E) Comparison of changes in the stem cell marker gene *Pdgfr, Nt5e, LepR* after HO modelling compared to controls in single-cell sequencing data. (F) Distribution of *Ctsk+* cells versus *Scx+* cells in TDMSC before and after injury. (G) Schematic diagram of the *Ctsk+* cell lineage-traced mice. (H) Schematic diagram of the Burn/ATP injury modeling of the Achilles tendon. (I) MicroCT scanning images and masson immunohistochemical staining of the ectopic ossification formation 9 weeks after sham surgery and injury modeling. (J) Data analysis of the HO bone volume, comparing the sham surgery group and the Burn/ATP injury modeling group by independent samples t test (n=6/group, **P<0.01). (K) Quantification of PC1^+^ cells and PC1^+^YFP^+^ double-positive cells in the x40 magnification field of view, comparing the mobile and immobilized groups by independent samples t test (mobile group n = 6, immobilized group n=4, ***P < 0.001). (L) Schematic diagram of a cell stretching experiment. Cells were stretched at 10% deformation rate, 0.5 HZ for one week, 4 hours per day. And qPCR experiments were performed on the mechanoreceptor gene *Pkd1*, the osteogenic gene *Alp*, and the fibrotic gene *Col3.*

**Supplemental Figure 2.** (A) Distribution of t-SNE cell populations from scRNA data of the Sham surgery group. (B) Distribution of t-SNE cell populations from scRNA data of the HO surgery group. (C) Distribution of t-SNE cell populations from scRNA data of the HO Immobile surgery group. (D) Analysis of scRNA data from mobile and immobile treated samples after HO modelling and analyze the expression of the stem cell marker gene *Pdgfra, Nt5e, LepR.* (E) Analysis of osteogenic and fibrogenic gene expression in *Pkd1+* versus *Pkd1-* cells. And analysing the effects of mobile and immobile interventions in *Pkd1+* cells on osteogenic and fibrogenic genes. (F) Analysis of osteogenic and fibrogenic gene expression in *Taz+* versus *Taz-* cells. And analysing the effects of mobile and immobile interventions in *Taz+* cells on osteogenic and fibrogenic genes. (G) Immunofluorescence staining image of Ctsk-YFP and Pdgfra depicting the periosteum and medullary cavity, both periosteal stem cells (red arrow) and osteoclasts (yellow arrow) were observed to be labeled.

**Supplemental Figure 3**. (A, B) Primary TDMSC were extracted for osteogenic induction cell culture and divided into four groups: control, addition of DAPT, addition of Notch pathway specific inhibitor IMR-1, and addition of both DAPT and IMR-1. ALP staining and qPCR were performed to detect osteogenic activity. (C, D) Primary TDMSC were extracted for osteogenic induction cell culture and divided into four groups: Control, addition of DAPT, overexpression of membrane-conjugated PC1-CTT, and overexpression of membrane-conjugated PC1-CTT together with the addition of DAPT. ALP staining and qPCR were performed to detect osteogenic activity. (E, F) Separation of cytoplasmic and nuclear proteins of TDMSCs after PKD1 siRNA interference to detect the nuclear translocation of TAZ, compared with Si NC group.
